# Why the Treatment of Mental Disorders Is an Important Component of HIV Prevention among People Who Inject Drugs

**DOI:** 10.1155/2013/690386

**Published:** 2013-01-17

**Authors:** Elizabeth Buckingham, Ezra Schrage, Francine Cournos

**Affiliations:** ^1^Substance Abuse and Mental Health Services Administration, 1 Choke Cherry Road, Rockville, MD 20857, USA; ^2^School of General Studies, Columbia University, 2970 Broadway, New York, NY 10027, USA; ^3^Mailman School of Public Health, Columbia University, 722 W. 168th Street, New York, NY 10032, USA

## Abstract

People who inject drugs are more likely to be HIV positive and to have a mental disorder than the general population. We explore how the detection and treatment of mental illness among people who are injecting drugs are essential to primary and secondary prevention of HIV infection in this population. Aside from opioid addiction, few studies have been conducted on the links between mental disorders and injection-drug use. However, independent of the injection-drug use literature, a growing number of studies demonstrate that untreated mental illness, especially depression and alcohol/substance use disorders, is associated with HIV-related risk behaviors, acquiring HIV infection, failure to access HIV care and treatment, failure to adhere to HIV care and treatment, and increased morbidity and mortality from HIV-related diseases and comorbidities. In our review of both the published literature and gray literature we found a dearth of information on models for providing care for both opioid addiction and other mental illnesses regardless of HIV status, particularly in low- and middle-income countries. We therefore make recommendations on how to address the mental health needs of HIV-positive people who inject drugs, which include the provision of opioid substitution therapy and integrated mental health, substance abuse, and HIV services.

## 1. Introduction

The HIV epidemic is intertwined with many other epidemics such as hepatitis C and tuberculosis and, just as powerfully, with substance use disorders and other mental illnesses. Throughout the world, HIV begins its spread among three vulnerable populations with high rates of mental disorders: people who inject drugs (PWID), men who have sex with men (MSM) and sex workers [1]. Following infection, the presence of HIV in the brain, HIV-related central nervous system and systemic complications, and the side effects of antiretroviral medications cause their own neuropsychiatric complications, further complicating this picture.

While in many treatment settings and government agencies substance use and other mental illnesses have been separated, this separation has created barriers to holistic care because there is considerable comorbidity between these two sets of disorders with nonaddictive mental disorders often preceding substance use disorders [2]. Data shows that among individuals with a lifetime history of substance abuse, over half were also affected by another mental disorder [3]. Conversely, individuals with nonaddictive mental disorders are more likely to have substance-use disorders than the general population. One survey found that 24% of individuals with lifetime major depressive disorder also had a substance-use disorder [3]. In a US national comorbidity study, aggregate analyses demonstrated significant prospective risks posed by baseline mental disorders for the onset of nicotine, alcohol and illicit drug dependence with abuse over the follow-up period [2].

This paper is focused on the key population of people who inject drugs keeping in mind that people engaging in injection-drug use overlap with both MSM and sex workers. We explore how the detection and treatment of mental illness among people who are injecting drugs are essential to primary and secondary prevention of HIV infection in this population. The term mental disorders will be used in this paper in a manner that is consistent with the Diagnostic and Statistical Manual of Mental Disorders Fourth Edition (DSM-IV) of the American Psychiatric Association [4].

Aside from opioid addiction, few studies have been conducted on the links between mental disorders and injection-drug use. However, independent of the injection-drug use literature, a growing number of studies demonstrate that untreated mental illness, especially depression and alcohol/substance use disorders, is associated with HIV-related risk behaviors, acquisition of HIV infection, failure to access HIV care and treatment, failure to adhere to HIV care and treatment, and increased morbidity and mortality from HIV-related diseases and comorbidities [5, 6]. In addition, with an increased focus on antiretroviral treatment (ART) as a strategy for primary and secondary HIV prevention, addressing mental disorders that interfere with regularly taking HIV medications is essential to treatment as prevention efforts.

Studies not only show the negative impact of mental disorders on HIV/AIDS, but also the positive impact of mental health treatment for mental disorders. For example, several studies have shown that antidepressant treatment is associated with improved adherence to antiretrovirals in HIV-positive individuals with depression [7, 8].

Yet the treatment of mental disorders remains a highly underfunded component of HIV prevention, care and treatment, even in many high-income countries. If treatment as prevention is to be effective, we must do better at identifying people who have HIV infection, linking them to care, retaining them in care, initiating antiretroviral treatment and achieving adherence that results in suppressed viral load. For example, the CDC approximates that the U.S. has an estimated 1.2 million persons of whom about 80% know that they are infected [9]. Of those who knew their status about three quarters were linked to care and half remained in care. Among all HIV-infected persons in the United States the CDC has estimated that only 28% are on antiretroviral treatment and have a suppressed viral load. We know that mental disorders have a negative impact at each step in the cascade of HIV testing, care, and treatment. We argue that the time has come to identify and treat mental disorders as an integral part of HIV prevention strategies for people who inject drugs and other overlapping vulnerable populations.

## 2. Epidemiology of HIV and Access and Adherence to HIV Care and Treatment among People Who Inject Drugs

People who inject drugs are becoming more important in the global landscape of HIV/AIDS because PWID are accounting for a larger proportion of new cases of HIV infection [10]. Injection-drug use has been identified in 148 countries with the largest numbers of injectors identified in China, the United States, and Russia [11].

Data on the extent of injection-drug use is absent for many countries in Africa, the Middle East, and Latin America. Africa, the continent with the largest HIV epidemic, has had an increasing role in global drug trafficking routes, leading to increasing injection-drug use transmission of HIV [12]. This is especially true in Nigeria, Kenya, Tanzania, South Africa, and Mauritius. Kenya alone had 7% of its new HIV infections attributable to drug injection in 2008, and 42% of Kenya's PWID population are HIV infected [12].

It is estimated that outside of sub-Saharan Africa, one in three new HIV cases is attributed to injection-drug use [10]. And in parts of the world where the epidemic is growing rapidly, such as Eastern Europe and Central Asia, some estimates show more than 80% of HIV transmission occurring among PWID [10]. People who inject drugs are more likely to be HIV positive than the general population. Estimates vary widely on the prevalence of HIV among PWID; however a 2008 systematic review suggests that the average global prevalence rate of HIV among PWID is 18% [8], compared to 0.8% of the general population [13].

In recent years HIV prevention efforts have increasingly incorporated the concept of treatment as prevention in response to a growing understanding that antiretroviral treatment can protect HIV-negative people from acquiring HIV and HIV-positive people from transmitting HIV. Because people who inject drugs play such a central role in the acquisition and transmission of HIV infection through risky sexual and drug use practices, it is very important for PWID to have access to and adhere to antiretroviral treatment. However, multiple studies have shown that even in settings where ART is widely available, people who inject drugs are less likely to start ART than other HIV-positive populations [3, 13]. Studies have found that PWID commonly present for HIV treatment later in the stage of the disease when AIDS symptoms are present. Later HIV-stage initiation of ART predicts worse prognosis and survival outcomes [3] and, as already noted, poses a greater likelihood of HIV transmission to others. PWID have also been found to be less adherent to antiretroviral treatment than other HIV positive populations [3, 14] and are more likely to discontinue ART treatment outright after it has been started [14].

## 3. Epidemiology of Mental Disorders among People Who Inject Drugs

The literature on mental illness among people injecting drugs is limited. The presence of substance use disorders among PWID is the most obvious link between psychiatric illness and injection-drug use. In the USA and elsewhere, the most commonly injected drugs are opioids and stimulants [15]. While injection of these substances could be an intermittent behavior that is not occurring in the context of addiction, studies of current and former people who injected drugs have found that the vast majority of them suffer from one or more addictive disorders; they are polydrug users, using both injected and noninjected substances, including alcohol. In addition, the presence of nonaddictive mental disorders was much higher than that seen in the general population.

While not focused on specific mental disorders, one study in Australia measured the self-reported wellbeing of the PWID participants using the Personal Wellbeing Index (PWI) and found that PWID had profoundly lower scores than the general Australian population [16]. A review of psychiatric comorbidity among people who inject drugs in Africa and Asia found that psychological distress was high, including suicidal ideation and attempts [17].

A team in Chicago [18] used various outreach techniques to gather mental health information on 570 young and mostly white PWID who were not currently in treatment and who had injected less than one month ago. About 98% of subjects met criteria for opioid dependence. Rates of alcohol, cannabis and cocaine abuse and dependence among male and female subjects varied between 14%–21%. Lifetime prevalence for other mental illnesses was much higher than in the general population and differed by gender: primary major depression: men 9%, women 21%; substance-induced major depression: men 22%, women 35%; posttraumatic stress disorder (PTSD): men 6%, women 17%; any primary anxiety disorder: men 13%, women 30%; antisocial personality disorder: men 30%, women 23%; borderline personality disorder: men 19%, women 28%.

It can be difficult to distinguish between psychiatric syndromes induced by drug intoxication/withdrawal from those that are independent of drug use. Brooner et al. attempted to do this by assessing 716 opioid abusers in Baltimore, Maryland after they had been stabilized on methadone maintenance [19]. Using the Structured Clinical Interview for DSM-III-R [20], the authors found that 24% of subjects met lifetime criteria for an Axis-I nonsubstance psychiatric disorder, most commonly major depression. On Axis-II, 35% had a personality disorder, most commonly antisocial personality disorder. At the time of entry into treatment 100% of subjects met criteria for current opioid dependence. In addition, lifetime rates of drug dependence with other substances were as follows: cocaine, 65%; cannabis, 51%; alcohol, 50%; sedatives, 45%; stimulants, 19%; and hallucinogens, 18%.

Other studies in the USA and Taiwan underscore the well-established finding that the vast majority of people who inject drugs suffer from addictive disorders [21, 22].

The type of drug addiction affects the degree of HIV risk that drug injection poses. For example, to maintain a high, individuals injecting cocaine, which is rapidly metabolized, must inject much more frequently than those using longer acting drugs like opioids; therefore obtaining new needles for each injection can become difficult [15].

Nonaddictive mental illnesses can also increase unsafe injection-drug use practices. In a study of 343 opioid-dependent adults recruited from 12 sites across the United States and enrolled in multisite studies of the National Drug Abuse Treatment Clinical Trials Network (CTN001-002), depressive symptoms were associated with an increased level of injection risk behaviors [23]. Past suicide attempts were associated with a history of drug injection among almost 7,000 Swedish criminal justice clients with suspected substance-related problems [24].

Although not specific to injection-drug use, the US national comorbidity study (NCS) of 5,000 people interviewed at two time points about a decade apart provides evidence that strongly suggests nonaddictive mental disorders often precede addictive disorders [2]. Among participants who had never used illicit drugs before, having any sort of psychiatric disorder almost tripled the risk for eventually using, abusing, or becoming dependent on illicit substances, and the greater number of psychiatric disorders the greater the risk. With regard to specific disorders and the risk of developing a future substance use disorder, major depression was associated with twice the risk; panic disorder, intermittent explosive disorder and oppositional defiant disorder with three times the risk; and attention deficit hyperactivity disorder and separation anxiety with about a fourfold increase in risk. The authors state “retrospective and prospective studies both indicate that mental disorders have a temporally primary age of onset in the majority of these forms of comorbidity”.

In summary, nonaddictive mental disorders often precede addictive disorders and both contribute to the risk of acquiring and transmitting HIV among people at risk for or currently injecting drugs, including high-risk sex, non-help-seeking behaviors, and nonadherence to ART.

## 4. Overlap between Injection-Drug Use, Sex Work, and Mental Illness

Injection-drug use and commercial sex work are strongly linked. Examining this link is helpful for two reasons: risky sexual behavior is an important route of HIV transmission from PWID to the general population and there is a larger literature on mental illness among sex workers than there is among those who inject drugs.

Commercial sex work is an economic exchange in which specific sexual activities are purchased or traded for other goods. Many social and economic factors are associated with sex work, including extreme poverty, illiteracy, and unaddressed (or even sanctioned) violence, especially against men who have sex with men and women. These factors are in turn associated with poor mental health. Multiple studies of sex workers, including female, male, and transgendered sex workers, have shown that this is a population with high rates of both addictive and nonaddictive mental disorders, as well as high rates of HIV-related risk behaviors.

In a study in St. Petersburg (Russia), 81% of surveyed sex workers said they injected drugs at least once a day, 65% of those injecting had used nonsterile injecting equipment, and 48% of sex workers were HIV-positive [25]. Similar rates of HIV infection were reported among female sex workers who inject drugs in Ho Chi Minh City (Viet Nam) [25]. Among men who inject drugs in Viet Nam, having contacts with female sex workers was associated with a greater likelihood of being HIV positive [26]. Illegal drug use, particularly with injection drugs, was the single greatest risk factor for HIV infection among female sex workers in Kaiyuan City, China [27].

A study in Zanzibar looked at 509 men who have sex with men (MSM) living in the community of whom 66 also injected drugs [28]. MSM-PWID were twice as likely to have HIV infection as non-PWID MSM. They were also five times less likely to wear a condom with a paid female partner and ten times less likely with a nonpaid female partner. MSM-PWID were much more likely to have engaged in group sex with other men in the past month. They also reported poor needle habits with a majority indicating that they used a needle after someone else and had passed around a needle after using it themselves. This study demonstrates that while MSM are at high risk of HIV acquisition and transmission, those who are also injecting drugs are at even higher risk, in part through unsafe drug injection practices and in part through links to commercial sex work and other risky sexual practices.

Transition to injection-drug use was associated with involvement in sex work among Aboriginal people in Canada [29], and in another Canadian study, this time of HIV-infected PWID who had achieved viral suppression on ART, sex-trade involvement was associated with viral rebound [30].

A study in Puerto Rico found that 47% of female sex workers injected drugs [31]. In this study sex workers with high levels of depressive symptoms had a 70% HIV infection rate, whereas those with low depressive symptoms had a 30% infection rate. This did not appear to be a consequence of HIV infection, since depressive symptoms were independent of HIV status.

Childhood sexual and physical abuse histories are common among male and female sex workers [32–35]. Moreover, throughout the world, including Australia, South Africa, Thailand, Turkey, the United States, and Zambia, sex workers report being raped and physically assaulted during the course of their work. These childhood and adult traumas are associated with significant suicidal ideation and risk, and high rates of mental disorders, especially depression and PTSD [16, 32–40].

Roxburgh et al. studied 72 female street-based sex workers in Australia. More than 80% were heroin dependent and injecting drugs [35]. About half of these sex workers had begun injecting drugs prior to sex work and used sex work to pay for their drugs. Half of the women reported using drugs to facilitate their sex work largely through their numbing effects. Among these women, 47% met a lifetime DSM-IV diagnosis of posttraumatic stress disorder. What emerged was a complex intertwinement of childhood abuse and neglect, PTSD, symptoms of depression, injection drug use, and engaging in sex work.

Vanwesenbeeck [41] conducted an exhaustive review of the research literature on sex workers from 1990 through 2000 and concluded that in the Western world, injection drug use and noncommercial sexual activity are the most important risk factors for HIV infection in female sex workers. This is similar to the findings of Gilchrist et al. [42] who studied 118 women who inject drugs in Barcelona and found that coercion into sex and sex exchange were common. However, noncommercial partners had a stronger influence on risky behaviors, needle sharing, and unprotected sex than did male clients.

## 5. Mental Disorders as Risk Factors for Injection-Drug Use

Mental disorders often precede the onset of injection-drug use. Again, the most obvious link is to current addictive disorders as already described above. In addition, studies show that early onset of alcohol and polysubstance use is an important risk factor for injecting drugs in adulthood [43].

Injection-drug use has been found to be a risk factor for HIV among people with severe mental illness. Meade and Sikkema [44] did a literature review of this link and found 17 studies demonstrating an average rate of ever injecting drugs of 22%, and a past year rate of 4%. Of these studies, eight looked at needle sharing and found on average 61% lifetime and 50% past year rates of this behavior.

## 6. Need for Mental Health Services for People Living with HIV/AIDS

The need for mental health services for people living with HIV/AIDS (PLWHA) is clear. Mental illness has been shown to impact HIV transmission as well as disease progression [45]. Depression, substance use disorders, and other severe mental illnesses are associated with transmission risk behaviors such as risky sexual activity and drug injection among both HIV-negative and HIV-positive people [46]. In terms of disease progression, poor mental health in general, and depression and substance use in particular, have been shown to negatively affect antiretroviral therapy adherence, which can cause poorer health outcomes and increased risk for HIV transmission [47, 48].

People who have been diagnosed with severe mental illnesses have a higher prevalence of HIV than the general population [49]. One study found individuals with serious mental illnesses 8 times more likely to be HIV positive than the general population [8]. Conversely, PLWHA have higher rates of mental disorders [14], particularly depression [50], with HIV-positive individuals nearly twice as likely to be diagnosed with major depression as HIV-negative individuals [50]. Depression is the most common mental disorder among HIV-infected people and is present in 30–50% of patients in HIV care and treatment settings [48]. Studies have found that chronic depressive symptoms are associated with a higher risk of mortality, particularly for women living with HIV, who are twice as likely to die as HIV-infected women with limited or no depressive symptoms [51, 52]. There is also evidence that psychological distress can shorten the time from HIV to AIDS, particularly among PWID [53].

## 7. Recommendations for Mental Health Services for People Living with HIV/AIDS

There are a variety of guidelines for addressing mental disorders among people with HIV/AIDS. We have chosen to present guidelines developed by the US Agency for International Development (USAID) because USAID has a global focus and has been very involved, particularly in their collaboration with the US Office of the Global AIDS Coordinator, in supporting the care of people with HIV/AIDS.

Support and Technical Assistance Resources (AIDSTAR-One) through USAID produced a technical briefing in 2009 on the mental health needs of people living with HIV/AIDS who suffer from mental health or substance use disorders [54]. They break down the continuum of HIV/AIDS into three phases: pre-ART, ART, and the advanced disease/end-of-life phase. Even as the pre-ART phase becomes briefer and in some countries disappears as patients who test HIV positive are offered immediate ART, the AIDSTAR-One recommendations remain relevant.

### 7.1. Pre-ART Phase

During the pre-ART phase of HIV/AIDS, PLWHA have five distinct mental health needs. Firstly, it is essential that PLWHA receive early screening for and diagnoses of mental health and substance use disorders. The mental disorders that are associated with HIV include adjustment disorders, mood disorders including major depression, anxiety disorders including panic disorder and posttraumatic stress disorder, substance use disorders, HIV-associated dementia (also known as AIDS dementia complex), and milder neurocognitive impairment. Treatment of co-occurring disorders can lead to improved HIV-related health outcomes and reduce transmission risk.

Secondly, PLWHA with a substance use or other mental disorder must have access to HIV care and treatment. PLWHA with co-occurring mental disorders are less likely to start and remain on ART treatment. Thirdly, when a person living with HIV/AIDS is first diagnosed, there are specific mental health needs that arise in response to the HIV diagnosis. HIV diagnosis can trigger concerns over death, stigma of the disease, changes in personal relationships, and uncertainty of the future. These concerns can trigger anxiety or depression and both can have a negative impact on accessing ART treatment or remaining in treatment. Fourthly, there are very specific mental health needs for PLWHA around stigma and discrimination as a result of being HIV positive. The consequences of HIV stigma can include social isolation, marginalization and discrimination and can directly impact a person's care-seeking behaviors. Finally, because HIV infection is a chronic illness, PLWHA are in need of ongoing psychosocial support. Psychosocial support can help reduce the psychological distress of living with HIV and also improve ART adherence and disease outcomes.

### 7.2. Antiretroviral Treatment Phase

During the ART phase, many of the mental health needs from the pre-ART phase are still relevant, particularly those dealing with stigma, access to care, emotional response, and psychosocial support. However there are certain mental health needs that are specific to the ART phase of HIV/AIDS in people with co-occurring disorders. These include adherence to ART, management of mental and substance use disorders, and side effects and cognitive impairments as a result of HIV itself and its treatment.

### 7.3. Advanced Disease/End-of-Life Phase

Despite the fact that effective treatment exists for HIV, many deaths still occur as a result of HIV infection. If a person living with AIDS progresses to very advanced stages of the disease where death is likely, mental health needs are particularly important in caring for that person and his or her caregivers. Both the patient and the caregivers will need substantive physical, emotional and spiritual support as they go through the process of death and dying, as well as grief and loss. These support systems can be both formal and informal.

## 8. Models for and Barriers to Mental Health Services for People Living with HIV/AIDS

It is currently not possible to carry out most of the AIDSTAR-One recommendations in most parts of the world, and the vast majority of people who inject drugs are not in treatment for their addictive or nonaddictive mental illnesses. While mental disorders are common, accounting for 13% of the total global burden of disease, adequate treatment is often not available [14]. In high-income countries, there is a 35–50% treatment gap for mental disorders. In low- and middle-income countries the gap is even more pronounced, with between 76–85% of those in need of services not receiving treatment for mental disorders [14]. Low- and middle-income countries have not only the largest treatment gap for mental disorders, but also the highest burden of HIV/AIDS, with sub-Saharan Africa alone accounting for 69% of the entire population of people living with HIV/AIDS [55]. Still another barrier to treatment is that drug use disorders are often criminalized rather than treated [56]. With the clear association between mental disorders and HIV/AIDS and the enormous gap in mental health treatment services, especially in low- and middle-income countries, there is an obvious need for improved HIV and mental health interventions.

In addition, virtually every funding stream for people with HIV/AIDS spends very little money on the treatment of mental disorders. Factors that explain this include the lack of recognition and the stigma of mental illnesses and the low priority given to these disorders by country leadership (both public and private), including in the USA, and by the HIV experts and scientists who drive the HIV treatment and research agenda. They may share a common misperception that mental health services are costly, derived from basing their estimates on per capita costs of psychiatric care, even though many services are now provided by community workers through an approach called task-shifting [57].

Another concern in relation to the specific needs of people who inject drugs is the availability and legality of opioid substitution therapy (OST). Of the five countries that have megaepidemics of HIV among PWID—Russia, China, Ukraine, Vietnam, and Malaysia—OST is available in all but Russia. In Russia, methadone and buprenorphine remain illegal for use in addiction treatment [58]. However, even in those countries where opioid substitution therapy is legal, OST is offered to less than 5% of patients that are in need of drug treatment. While these percentages are very low, they are on the rise and countries, particularly those with megaepidemics, with the exception of Russia, are increasing the number of people receiving OST [58].

In high-income countries, OST is generally more available. By 2000, all but two European Union countries (Cyprus and Estonia) had introduced opioid substitution therapy, providing drug treatment for approximately one third of the PWID. It is estimated that between 1998 and 2004, 15% to 25% of addicted opioid users were receiving opioid substitution therapy [59].

While availability of OST is often the first step to treating PWID, maintaining drug treatment is also important. In the Mackesy-Amiti et al. [18] US-based study of PWID, while 68% had received some form of substance abuse treatment in the past, only 5% were currently in treatment and only 10% reported current 12-step program attendance.

Beyond the treatment of opioid addiction with OST and the provision of clean injection equipment, it is difficult to identify effective and tested treatment models for HIV positive PWID with the common psychiatric comorbidities that we have discussed. Our own search through the Columbia University Libraries article database and PubMed revealed a dearth of peer-reviewed articles on models for providing care for both opioid addiction and other mental illnesses regardless of HIV status. Rarer still are articles that concern non-Western countries, as other reviewers have noted [17]. Even fairly comprehensive articles on the subject of illicit drugs and HIV treatment seem to handle mental illness as merely another comorbidity that includes its own set of prescription drugs with specific interactions, rather than addressing the absence of systems to deliver mental health care [60].

We therefore also searched the gray literature in the hopes of finding studies, technical reports, and other publications that could aid the search for information about active treatments in the field. Some of the largest organizations with the greatest reach financially and logistically do not include much information on mental health in their reports. Organizations and programs like UNAIDS, the World Health Organization, and the US Government's President's Emergency Plan for AIDS Relief (PEPFAR) often mention mental health briefly and in terms of the broader psychosocial aspects without going into psychiatric diagnoses and the options to treat them [10, 61–65].

Some smaller organizations have been making strides in treatment for HIV positive PWID and in reporting on methods of treatment. For example reports from the Global Initiative on Psychiatry are often in-depth and discuss the treatment options, or lack thereof, with a focus on mental health and HIV globally, in such places as Eastern Europe [66], Kazakhstan [67], and Tajikistan [68]. If there is to be any progress in the integration of mental health treatment options for HIV positive PWID, it will take a concerted effort on the part of all organizations to include and incorporate the screening for and treatment of mental disorders into their activities.

Extrapolating from the existing literature on treating comorbid substance use and nonaddictive mental disorders, a number of recommendations can be made for meeting the mental health needs of people who inject drugs. To the extent possible it works best to have one-stop care where all services are integrated within the same program and team meetings take place with all providers present [69]. This would include the integration of medical care for those programs that serve HIV positive populations. Use of a shared electronic record in this setting further enhances integration because providers can rapidly see one another's interventions. Mental health services that can be provided in integrated settings include comprehensive assessment and differential diagnosis; medications for both psychiatric and substance use disorders; psychotherapies, especially motivational interviewing and cognitive behavioral therapy; psychosocial support and social services; a thorough assessment of drug interactions and toxicities; and a comprehensive way to monitor people with multiple chronic relapsing disorders.

Other possibilities to achieve at least some degree of integration of services, in descending order of the likelihood of success, are programs colocated at the same site even though they are not integrated, case managers who escort patients from one service to other unrelated services, and having clinicians in unrelated settings share information and clinical decision making. Because buprenorphine treatment is more realistic to provide than methadone in medical settings, use of this agent for opioid substitution therapy can also facilitate integration of services. Screening consenting clients for common mental illnesses in methadone maintenance programs and those that offer clean injection equipment could help facilitate comprehensive care if such programs are linked with and make referrals to mental health services.

Achieving integrated services will require more successful strategies for funding the treatment of mental disorders, especially in low and middle-income countries; new approaches to overcoming systemic barriers to integration, such as the tendency seen in the United States to separately fund and operate services for addictive and nonaddictive mental disorders [70]; and a commitment by the largest global health care organizations to include the diagnosis and treatment of mental disorders in HIV/AIDS and other treatment guidelines and programming.

## 9. Conclusion

People who inject drugs are more likely to be HIV positive and to have a mental disorder than the general population. The health needs of PWID should be met not only because it is a human right, but also to effectively combat the global HIV epidemic. In order to address the needs of HIV positive PWID, policy and programs must include mental health services, which include adequate and available drug treatment options, including opioid substitution therapy. Addressing the mental health and substance abuse needs of people who inject drugs will help to prevent the acquisition and spread of HIV. Mental disorders and injection-drug use are highly stigmatized and remain a low priority for HIV-related funding and research. More research must be done to understand the relationship between mental disorders, injection-drug use and HIV prevention, and more programming addressing these three problems in a comprehensive way needs to be developed, funded, and studied.
